# Incidence, Etiology, and Healthcare Utilization for Acute Gastroenteritis in the Community, United States

**DOI:** 10.3201/eid2811.220247

**Published:** 2022-11

**Authors:** Mark A. Schmidt, Holly C. Groom, Andreea M. Rawlings, Claire P. Mattison, Suzanne B. Salas, Rachel M. Burke, Ben D. Hallowell, Laura E. Calderwood, Judy Donald, Neha Balachandran, Aron J. Hall

**Affiliations:** Center for Health Research, Kaiser Permanente Northwest, Portland, Oregon, USA (M.A. Schmidt, H.C. Groom, A.M. Rawlings, S.B. Salas, J. Donald);; Centers for Disease Control and Prevention, Atlanta, Georgia, USA (C.P. Mattison, R.M. Burke, B.D. Hallowell, L.E. Calderwood, N. Balachandran, A.J. Hall);; Cherokee Nation Assurance, Arlington, Virginia, USA (C.P. Mattison, N. Balachandran);; Oak Ridge Institute for Science and Education, Oak Ridge, Tennessee, USA (L.E. Calderwood)

**Keywords:** gastroenteritis, United States, enteric infections, bacteria, food safety, fungi

## Abstract

Knowledge of the epidemiology of sporadic acute gastroenteritis (AGE) in the United States is limited. During September 2016–September 2017, we surveyed Kaiser Permanente Northwest members in Oregon and Washington, USA, to collect data on the 30-day prevalence of dually defined AGE and diarrhea disease and related health-seeking behavior; from a subset of participants, we obtained a stool specimen. Using the iterative proportional fitting algorithm with raked weights, we generated AGE prevalence and annualized rate estimates. We detected norovirus, rotavirus, astrovirus, and sapovirus from submitted stool specimens through real-time quantitative reverse transcription PCR (qRT-PCR). We estimated a 30-day prevalence of 10.4% for AGE and 7.6% for diarrhea only; annual rates were 1.27 cases/person/year for AGE and 0.92 cases/person/year for diarrhea only. Of those with AGE, 19% sought medical care. Almost one quarter (22.4%) of stool specimens from those reporting AGE tested positive for ≥1 viral pathogen, compared with 8.2% from those without AGE.

In the United States, the incidence of acute gastroenteritis (AGE) is high. AGE is estimated to cause 179 million illnesses annually (*1*,*2*). Precise data are limited on the occurrence and characteristics of sporadic AGE, particularly because the illnesses are generally mild and usually do not require medical care; may not have had diagnostic testing even if care was sought; and, depending on the pathogen, may not be reportable through public health surveillance systems. Previous US publications, using data from the US Foodborne Diseases Active Surveillance Network (FoodNet), have reported AGE prevalence ranging from 7.7 to 11%, equivalent to roughly 0.7–1.4 illnesses/person/year, depending on the recall period (i.e., 7 or 28 days) and symptom profile (i.e., diarrheal illness alone or with the presence of additional symptoms) (*1*,*3*–*5*). These studies have been essential in establishing estimates of AGE incidence in the community and highlighting the substantial burden of disease. However, differences in AGE case definitions have complicated efforts to compare findings across studies and time periods, and robust estimates of occurrence across the age spectrum remain limited. Consequently, there is a need to obtain all-age, population-based estimates of AGE within the United States.

Even assuming the lowest reported AGE prevalence of 7.7%, there is potential for substantial disease burden on the local healthcare systems and on society, such as through lost productivity (*6*). Among persons with AGE, 12%–20% have reported visiting a healthcare provider to manage their symptoms, and AGE has been estimated to contribute to 2–3 million ambulatory visits and 900,000 hospitalizations per year in the United States (*1*,*3*,*4*,*7*–*10*). However, these data have relied on samples of persons within a geographic area who may differentially seek care depending on if they have medical insurance or access to an affordable care source. As a result, these studies may not accurately estimate the true potential burden on a healthcare system.

Clarifying the etiology of AGE illness within communities and healthcare systems can help to effectively target prevention efforts. Sporadic cases of AGE are largely attributable to viral pathogens; norovirus is the most common cause of AGE across the age spectrum. Evidence in the literature suggests that intensity of viral shedding among those with asymptomatic norovirus infections is similar to that of symptomatic infections (*2*,*8*,*11*); however, according to transmission modeling of a healthcare-associated outbreak, symptomatic shedders are more likely to transmit norovirus to others than those without symptoms (*12*).

To better characterize the incidence of AGE in the community, the associated healthcare utilization, and the prevalence of viral enteropathogens among both symptomatic and asymptomatic persons, we conducted the Community Acute Gastroenteritis (CAGE) Study among the membership population of a large, integrated healthcare system. The aims of the CAGE Study were to generate 30-day prevalence and annualized incidence estimates of AGE occurrence across the age spectrum, describe the proportion of symptomatic persons seeking healthcare, and calculate the prevalence of enteric viral pathogens among those who did and did not report AGE. To contextualize our results with previously reported literature, we report our findings here using 2 validated case definitions (*1*,*13*).

## Methods

### Study Population

We conducted the CAGE Study within Kaiser Permanente Northwest (KPNW), an integrated health care delivery system with >600,000 current members. This network comprises 24% of, and is demographically similar to, the underlying population of northwest Oregon and southwest Washington, USA (*14*).

### Sampling and Recruitment

We targeted enrollment to ≈3,000 members of all ages over a 12-month period. To achieve this goal, we selected age-stratified, simple random weekly samples of KPNW members from September 26, 2016, through September 19, 2017. Sampling was conducted without replacement through automated abstraction of health plan enrollment records, updated monthly, and was unrelated to AGE illness status or healthcare utilization. We excluded members who were in hospice care, non-English speaking, decisionally or cognitively impaired, previously recruited for the study, or had opted out of all KPNW research activities. Within the randomly sampled population, we targeted enrollment of an age-stratified subset of 500 members to complete a survey and provide a stool sample for virologic testing (SS cohort); the remaining 2,500 targeted enrollees were asked to complete a survey only (SO cohort). Although we describe the prevalence of viral pathogens among both cohorts, we sampled 500 in the SS cohort to have adequate power to detect an estimated 5% prevalence of these pathogens among asymptomatic persons.

Every week, we first invited our selected sample to participate by mailing recruitment postcards containing information about the study and a link to an online survey. Three days later, we sent recruitment email invitations to sampled members with active email addresses on file; we sent a reminder email invitation 1 week later. For participants within the SO cohort, we made no further recruitment efforts. To sampled members within the SS cohort, study staff made recruitment phone calls beginning 1 week after email invitations were sent; staff made >3 phone call attempts over the course of 1 week.

To compensate for their time, we provided enrolled participants who completed only the survey (all SO participants and those SS participants who did not provide a stool specimen) a $10 gift card. We compensated SS participants who completed a stool specimen with a $20 gift card.

### Survey

The 34-item survey administered to participants comprised questions on demographic characteristics and about AGE symptoms in the previous 30 days. For those reporting AGE symptoms, we collected the frequency of vomiting/diarrhea for the most recent illness and information on any related medical encounters. We assessed encounter types separately and included inpatient hospitalizations; urgent care, emergency department, and outpatient visits; and telephone and email encounters. We defined telephone and email encounters as remote and defined the remaining encounter types as in-person. We also asked survey respondents to self-report medical conditions associated with the occurrence of chronic diarrhea as a major symptom (i.e., Crohn’s disease, ulcerative colitis, inflammatory bowel disease, or abdominal or colorectal cancer).

### Case Definitions

Our primary AGE case definition included participants who reported any vomiting (>1 episode within 24 hours) or diarrhea (>3 loose stools in any 24-hour period) (*3*). Participants with <3 loose stools in a 24-hour period and no vomiting were not considered to have AGE. Persons with medical conditions associated with chronic diarrhea were considered to have AGE if they reported vomiting; otherwise, they were categorized as noncases, regardless of diarrhea episodes.

For incidence and healthcare utilization analyses, we separately considered a second case definition limited to all persons reporting acute diarrhea, which we defined as having >3 loose stools in any 24-hour period (*15*). Participants with <3 loose stools in a 24-hour period, those reporting vomiting only, and those with medical conditions associated with chronic diarrhea were categorized as noncases.

### Stool Collection and Laboratory Testing

For SS participants, we employed the same method for stool sample self-collection as previously described for our medically attended acute gastroenteritis (MAAGE) study; stool sample kits were sent to responders by overnight courier within 1 day of survey completion (*14*). Once returned, the Oregon State Public Health Laboratory (OSPHL) conducted laboratory testing of stool specimens submitted by study participants to detect norovirus, rotavirus, astrovirus, and sapovirus, using TaqMan real-time quantitative reverse transcription PCR (qRT-PCR) protocols developed by the Centers for Disease Control and Prevention (CDC), also as previously described (*14*). OSPHL forwarded stool specimens testing positive for rotavirus to the CDC for confirmatory testing by qRT-PCR and enzyme immunoassay (EIA).

### Statistical Analyses

We conducted all analyses using weights to account for the age-stratified probability sampling. In brief, we calculated a base weight to account for the initial probabilities of selection within each age stratum and week of sample selection. For the SO sample, we calculated a nonresponse adjustment factor using 10 strata defined by age group (0–4, 5–17, 18–44, 45–64, and >65 years) and sex to reduce potential bias due to nonresponse; for the SS sample, we calculated the nonresponse adjustment factor using the 5 age strata. Last, we raked the weights using the iterative proportional fitting algorithm (*16*) so that the marginal totals by age and sex matched known KPNW population totals from September 2017, when the CAGE survey was completed. We obtained SEs using Taylor series linearization and calculated 95% CIs by using exact (Clopper-Pearson) formulas.

We report population characteristics using unweighted counts and weighted means or proportions. We estimated 30-day point prevalence with 95% CIs by using weighted proportions. We calculated prevalence estimates overall, by age group, and by month. For monthly estimates, we included responses to the survey occurring before the 15th of the previous month; we included responses that occurred on or after the 15th in the calculation of prevalence for the current month. Using the prevalence estimate, we then calculated an annualized rate by multiplying the prevalence by 365/30, which yields and estimate of the average number of AGE cases per person per year.

For prevalence and reported healthcare encounter estimates, we report calculations using the AGE and acute diarrhea case definitions. Because of the small sample of persons meeting the acute diarrhea case definition from among those submitting a stool specimen for virologic testing, we report results using only the AGE case definition. We conducted all analyses in Stata version 15.1 (https://www.stata.com).

### Ethics Statement

This project was reviewed and approved by the KPNW Institutional Review Board (FWA00002344). Participants provided informed consent to participate in this study.

## Results

**Figure 1:**
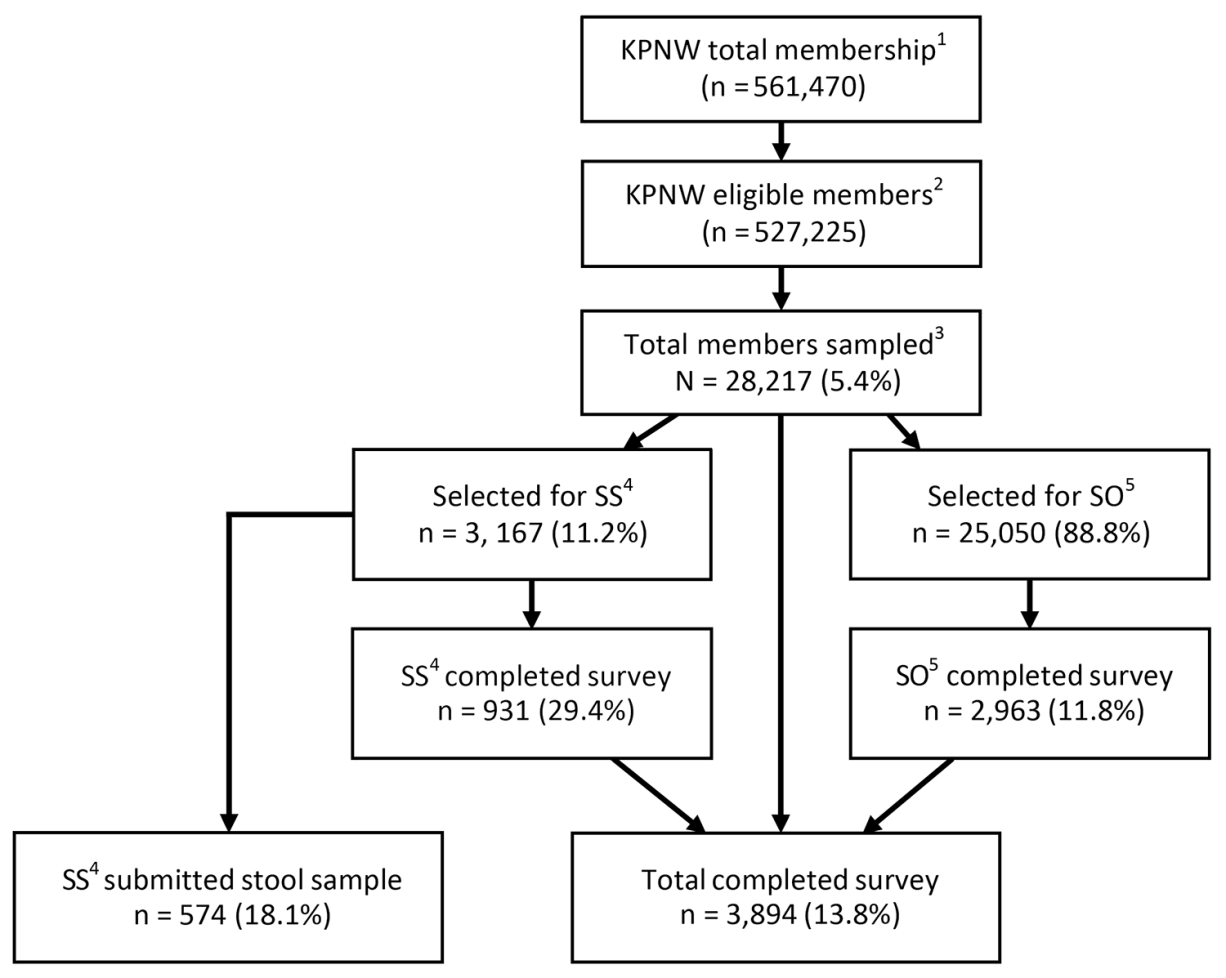
Sampling and inclusion of participants in Community Acute Gastroenteritis Study, Oregon and Washington, USA, September 2016–September 2017. KPNW membership as September 19, 2017; eligible members excluded those who were deceased, in hospice care, non-English speakers, decisionally/cognitively impaired, or opted out of all KPNW research activities. Sampling strategy was revised on April 10, 2017, to account for differences in response rates by age. AGE, acute gastroenteritis; KPNW, Kaiser Permanente Northwest; SO, survey only cohort, recruited to complete survey only; SS, stool sample cohort, recruited to complete survey and submit a stool sample.

In our 52-week study period, our sex- and age-stratified random sample comprised 28,217 KPNW members; 3,167 were selected for the SS cohort and 25,050 for the SO cohort. From this sample, we received a total of 3,894 surveys and 574 stool specimens. (Figure 1). On an unweighted basis, we observed a higher proportion of responses for those <5 years of age, >45 years of age, female, and of non-Hispanic ethnicity; we observed a lower proportion of responses among those 18–44 years of age (Table 1). After weighting, we observed a similar distribution across demographic characteristics between survey responders and the KPNW membership. The weighted mean age of participants was 40.1 years; weighted percentage by sex was 52% female and by race was 81% White (Table 1).

**Table 1:** Demographic characteristics of participants in Community Acute Gastroenteritis Study, Oregon and Washington, USA, September 2016–September 2017

Characteristic	Unweighted no.	Weighted %*
All participants	3,894	
Age group, y		
<5	473	4.8
5–17	334	14.6
18–44	951	38.0
45–64	1,283	27.3
>65	853	15.3
Sex		
M	1,542	
F	2,352	51.8
Race		
White	3,276	81.5
Other/unknown		
Ethnicity		
Hispanic	224	7.4
Non-Hispanic	3,261	87.6
Unknown/not specified	215	5.0
Education		
Less than high school	33	1.1
High school diploma/GED	320	10.7
Some college	942	30.8
College graduate	982	33.4
Postgraduate	752	24.0
Residence		
Urban	1548	40.7
Suburban	1555	41.8
Other	711	17.5
Insurance status		
Commercial only	2763	77.0
Medicaid	221	6.0
Medicare	871	15.9
Both Medicare and Medicaid	2	0.1
None	37	1.0
Income		
<$50,000	428	10.4
$50,000–$75,000	664	16.2
>$75,000	520	12.9
Missing/declined to state	2282	60.5

*Weighted to account for age-stratified probability sampling.

Overall, 395 participants met our primary AGE case definition, resulting in a 30-day AGE age-weighted prevalence of 10.4%, equivalent to a rate of 1.27 cases/person/year. Among those participants, 23% reported both diarrhea and vomiting, 50% reported only diarrhea, and 27% reported only vomiting. A total of 289 participants reported having acute diarrhea, resulting in a 30-day diarrheal prevalence of 7.6%, equivalent to a rate of 0.92 cases/person/year (Table 2). A total of 124 participants (3.2%) reported having had 1–2 loose stools but did not meet criteria for either case definition.

**Table 2:** Estimated 30-day prevalence and number of persons with AGE, by age group, in Community Acute Gastroenteritis Study, Oregon and Washington, USA, September 2016–September 2017*

Category	Case definition: diarrhea or vomiting			Case definition: acute diarrhea	
	AGE illness, unweighted no.	Prevalence (95% CI)		AGE illness, unweighted no.	Prevalence (95% CI)
By age group†					
<5	65	13.5 (10.6–16.9)		37	7.8 (5.5–10.6)
5–17	36	10.6 (7.5–14.5)		12	3.3 (1.7–5.8)
18–44	120	12.5 (10.3–15.0)		96	10.2 (8.2–12.6)
45–65	119	9.2 (7.6–11.0)		104	8.0 (6.5–9.7)
>65	55	6.4 (4.9–8.3)		40	4.7 (3.4. 6.3)
By sex‡					
M	153	10.1 (8.4–12.1)		106	7.5 (5.9–9.3)
F	242	10.7 (9.4–12.2)		183	7.8 (6.7–9.0)
Overall	395	10.4 (9.3–11.6)		289	7.6 (6.7–8.7)

*AGE episodes were defined based on self-report as any illness in the previous 30 day with diarrhea or vomiting that included >3 loose stools in any 24-hour period. Data from participants who completed the survey on or before the 15th of the month were included in estimates for the preceding month, whereas information from surveys completed after the 15th contribute to the current month. Prevalence estimates are weighted to account for the sampling scheme; 95% CIs are estimated using exact (Clopper-Pearson) formulas. AGE, acute gastroenteritis. †Survey design-based F-tests comparing differences in proportion of AGE across age groups are significant for both the diarrhea or vomiting definition (p = 0.0014) and the diarrhea definition (p<0.001). ‡Survey design-based F-tests comparing differences in proportion of AGE across sex are not significant for either the diarrhea or vomiting definition (p = 0.264) nor the diarrhea definition (p = 0.112).

**Figure 2:**
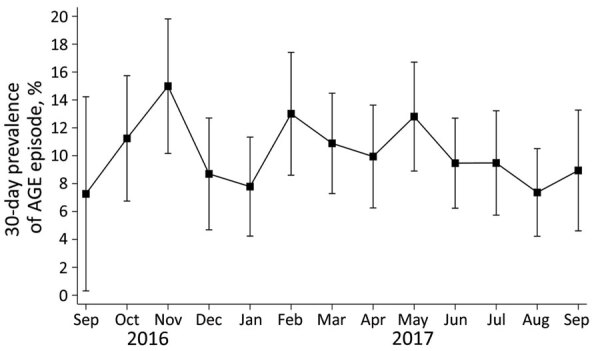
Estimated 30-day prevalence of AGE episodes by month, primary case definition, in Community Acute Gastroenteritis Study, Oregon and Washington, USA, September 2016–September 2017. AGE episode was defined based on self-report as any illness in the previous 30 days with diarrhea or vomiting that included >3 loose stools in any 24-hour period. Data from participants who completed the survey on or before the 15th of the month were included in estimates for the preceding month, whereas information from surveys completed after the 15th contributed to the current month. Prevalence estimates are unadjusted and weighted to account for the sampling scheme; 95% CIs are estimated using the delta method and normal approximations. AGE, acute gastroenteritis.

We observed no significant difference in prevalence estimates between male and female participants (p = 0.264). When examined by age, the prevalence of AGE illness was highest among the youngest age group (0–4 years, 13.5%) and lowest among the oldest (>65 years, 6.4%). For acute diarrhea, the highest prevalence occurred among those 18–44 years of age (10.2%) and those 5–17 years of age (3.3%). Those 0–4 years and 5–17 years of age had comparatively low prevalence (7.8% and 3.3%, respectively) when using the diarrhea-based definition, compared with a prevalence of 13.5% and 10.6%, respectively, when using the primary case definition (Table 2). We observed monthly variability in the occurrence of AGE, but we observed no statistically significant seasonal patterns (Figure 2).

### Healthcare Encounters

Overall, 80 (19%) persons with AGE had >1 AGE-related healthcare encounters within KPNW. Most of those (63 [79%]) had an in-person encounter, 37 (46%) of whom also had a remote encounter; 17 (19%) had only a remote encounter (Table 3). The percentage of participants seeking AGE-related medical care was slightly lower among persons reporting only acute diarrhea; 17% had >1 encounter overall, of which 77% had an in-person visit and 23% had a remote encounter only.

**Table 3:** Distribution of encounters and encounter types among persons with AGE in Community Acute Gastroenteritis Study, Oregon and Washington, USA, September 2016–September 2017*

Category	Case definition: diarrhea or vomiting			Case definition: diarrhea	
	Unweighted no. encounters	% Encounters (95% CI)		Unweighted no. encounters	% Encounters (95% CI)
No encounter	313	80.9 (76.0–85.1)		288	83.3 (77.7–87.9)
Any encounter, by type	80	19.1 (14.9–24.0)		51	16.7 (12.1–22.3)
Inpatient	10	12.2 (5.5–22.3)		5	9.2 (2.6–21.7)
Urgent care or emergency department	37	49.5 (35.8–63.1)		24	52.0 (34.1–69.6)
Outpatient	55	69.8 (56.4–81.2)		33	63.8 (44.9–80.0)
Remote	54	64.4 (50.4–76.9)		35	67.3 (48.3–82.9)
Any encounter, by delivery method	80	19.1 (14.9–24.0)		51	16.7 (12.1–22.3)
Remote only†	17	18.8 (10.8–29.4)		13	23.5 (11.5–39.7)
In-person only‡	26	35.5 (23.1–49.6)		16	32.7 (17.1–51.7)
Both in-person and remote	37	45.6 (32.1–59.6)		22	43.8 (26.2–62.3)

*Percentage estimates are unadjusted and weighted to account for the sampling scheme. AGE, acute gastroenteritis. †Email, video call, or call nurse contact without an in-person visit. ‡Inpatient, urgent care, emergency department, or outpatient visit without any remote contact.

### Pathogen Testing

**Figure 3:**
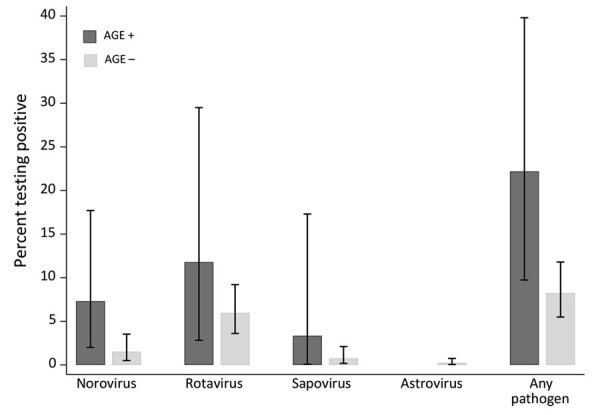
Viral prevalence by primary AGE case definition in Community Acute Gastroenteritis Study, Oregon and Washington, USA, September 2016–September 2017. Estimates are unadjusted and weighted to account for the sampling scheme; confidence intervals are estimated using exact (Clopper-Pearson) formulas. *Rotavirus results reported here reflect quantitative reverse transcription PCR testing results and not subsequent enzyme immunoassay test results (for which only 4 quantitative reverse transcription PCR positives were also enzyme immunoassay–positive). AGE, acute gastroenteritis.

In total, 574 SS study participants (with and without AGE) returned stool samples. On average, stool samples were collected within 10 days of survey completion; 83% of samples were collected within 2 weeks of survey completion. Of the samples, 570 (99%) were usable and tested at OSPHL, 70 collected from persons who met our primary definition for AGE and 500 collected from those who did not. As a weighted percentage, those totals yield an estimated 30-day AGE prevalence of 12.3% (95% CI 8.8%–16.5%) in this group. Overall, norovirus and rotavirus were the most commonly detected viral pathogens (Figure 3). Using this sample, we estimated that 22.4% (95% CI 9.6%–40.8%) of our total KPNW population with AGE would test positive for >1 viral pathogen. An estimated 7.2% (95% CI 2.0%–17.5%) would test positive for norovirus alone, 11.5% (95% CI 2.4%–30.1%) for rotavirus alone, 3.5% (95% CI 0.1%–19.0%) for sapovirus alone, and <1% for both norovirus and rotavirus. Using the sample of 500 specimens from persons without AGE, we estimate that 8.2% (95% CI 5.5%–11.7%) would test positive for >1 viral pathogen: 1.4% (95% CI 0.4%–3.4%) for norovirus alone, 5.8% (95% CI 3.5%–9.0%) for rotavirus alone, 0.98% (95% CI 0.32%-2.32%) for sapovirus alone, and 0.59% (95% CI 0.12%-1.74%) for astrovirus alone.

Of the 23 total stool specimens that tested positive for norovirus, 5 were determined to be genogroup I and 18 to be genogroup II. Forty of the 41 stool specimens (98%) testing positive for rotavirus by qRT-PCR at OSPHL were available for confirmatory testing at CDC. Of those, 1 (3%) was determined to be rotavirus vaccine shedding, and 3 (8%) asymptomatic persons tested positive by rotavirus EIA (however, all 3 had high Ct values, indicating lower viral load, and could not be genotyped).

## Discussion

Our study results confirm that use of an expanded case definition that includes persons reporting only vomiting increases prevalence estimates by ≈50% compared with a definition that includes only diarrhea (*13*). Our 30-day AGE prevalence estimates are broadly consistent with the range observed in previous literature, although the use of differing case definitions make direct comparisons more complex. Reporting our results in 2 ways improves our ability to compare our findings to those of others; however, doing so reduces the consistency observed between our prevalence estimates. For our AGE case definition, we observed a prevalence estimate of 10.4%, compared with Canada’s Foodbook estimate of 5.7% in 2014–2015 (*15*). For our diarrhea-only case definition, our estimate of 7.6% was lower than FoodNet’s estimate of 10%–11% from 1996–1999 (*1*,*3*,*4*). Those differences highlight the inherent variability in estimates of AGE, which may reflect variations in occurrence due to geography, time, or other factors.

Previous work has argued for use of the expanded AGE case definition (*7*). Excluding symptoms of vomiting has been associated with decreased sensitivity for identifying norovirus infections (*1*,*13*,*17*). Further, research has shown age-related differences in AGE symptom profiles, particularly with vomiting (*18*,*19*). For instance, 1 study found that vomiting was reported for 37% of AGE patients <5 years of age, compared with only 17% of those 65–74 years of age (*20*). This finding is further supported by our data, where we saw a lower prevalence of AGE among participants 0–4 years of age when we used the case definition that did not include vomiting (7.8%) compared with the definition that did (13.5%). Considering that norovirus is the most common cause of AGE among children younger than 5 years (*2*), use of an expanded AGE surveillance case definition will yield more complete estimates of norovirus burden.

Nearly 1 in 5 (19%) of our respondents sought medical care for their AGE symptoms, consistent with behaviors reported in other US studies, even where 8%–9% did not have insurance coverage (*1*). Our study is unique in describing AGE-related healthcare seeking behavior that includes not only telephone consultations with a clinician for illness management but also other remote encounters, such as email exchange via patient portal and video appointments. One previous FoodNet publication asked whether respondents made a call to a medical provider, but it is unclear whether the calls were used to discuss management of clinical symptoms (in place of in-person visits) (*3*). KPNW encourages members to use those remote technologies for initial access to medical providers to make healthcare more accessible; to reduce the burden of in-person visits to medical offices; and to reduce the risk for transmission of communicable diseases to other members, staff, and clinicians. Whereas most clinical care was in-person, 19% of our population exclusively used remote encounters. As healthcare delivery systems increasingly expand access to virtual care, more research is needed to determine how this shift affects the burden of AGE on the healthcare system. If our population were generalizable to the US population, there would be an estimated 415 million AGE illness episodes per year, with an associated 5.4 million in-person AGE-related encounters. Our novel findings of an extrapolated 1.2 million remote AGE-related healthcare encounters indicate a potential higher burden on the healthcare system due to AGE that has not been well-captured in previous studies.

We observed an overall pathogen positivity of roughly 10% from among all submitted stool specimens; differences observed in pathogen positivity between those who did and did not have AGE were not statistically significant. This finding is likely because of the small numbers within our SS cohort and the potential time lag between occurrence of symptoms and collection of stool sample, as well as the high rate of rotavirus detection by qRT-PCR. Further work in this area is needed, but our study was powered to calculate the prevalence of viral pathogens among asymptomatic persons, rather than to detect significant differences between those with and without symptoms of AGE. Among those not meeting the AGE case definition, we observed a norovirus prevalence of 1.4% and a rotavirus prevalence of 5.8% by qRT-PCR; both values are slightly lower than previously published estimates of 4% and 11%, respectively (*21*,*22*). The prevalence of rotavirus detection is higher when using qRT-PCR versus EIA testing, because of the increased sensitivity of PCR tests for detecting viral pathogens at a lower viral load (*23*), which is supported by our findings. Because lower levels of shedding may be less clinically relevant, EIA continues to be preferred for routine surveillance rotavirus testing (*24*). However, the detection of both norovirus and rotavirus from persons without recognized AGE highlights a potential reservoir for sporadic AGE within the community, although asymptomatic infections are believed to be less contagious. Even so, interventions designed to reduce the transmission of AGE-related pathogens are important to follow for both symptomatic and asymptomatic persons.

A key strength of our study is that participants were selected via an age-stratified, representative sample of KPNW enrollees, which is reflective of the underlying population base. Consequently, we have been able to more accurately calculate the estimated number of AGE episodes per person per year when compared with other studies, and our findings are generalizable to the target population of this area. Further, conducting this study within our population reduces the likelihood that access to care is a barrier to seeking treatment in the healthcare system. Conversely, our study may have been limited by a low overall participation rate of 13.8%. This percentage is higher than reported in a comparable study from the United Kingdom (*25*), and we exceeded our sample size goal by obtaining 3,874 completed surveys and 574 stool specimens; however, the percentage is lower than those for other published studies of comparable design, such as FoodNet (*1*). We attempted to minimize the effects of this bias by using weighting that incorporated a nonresponse correction factor to improve the generalizability of our findings. Although we recognize the potential for recall bias, because our findings are based on reports of AGE within the previous 30-days, previous work examining the effects of a 7-day versus 1-month recall period found no difference in monthly prevalence estimates (*5*). Therefore, we believe any effect on our findings will be minimal. Our study may also have been limited by a delay between survey completion and stool sample collection, resulting in an underestimate of the prevalence of viral pathogens among those meeting our AGE case definition during the 30-days before survey completion. Because our study was powered to detect the prevalence of viral pathogens among those asymptomatic for AGE, we believe the effects on our findings would have been minimal.

In conclusion, AGE continues to exert a substantial burden of disease within the population, as well as upon healthcare delivery systems. This effect is particularly notable when vomiting is considered as part of the AGE case definition; prevalence estimates were nearly 50% higher when including this symptom. Our findings also provide key estimates of the prevalence of asymptomatic shedding of AGE-related viral pathogens, which can help contextualize viral prevalence data from AGE cases to assess disease burden. General interventions designed to reduce the transmission of AGE-related viral pathogens (e.g., hand hygiene) continue to be crucial as a means to reduce the extent of AGE in the population, even among persons without symptomatic disease. However, the high number of AGE cases in the community, particularly when including vomiting-only symptoms, leads to a heavy burden on the healthcare system. Additional targeted interventions, such as vaccines, could help reduce AGE in the community and, thus, reduce strain on healthcare systems.
